# Characterizing Roles for the Glutathione Reductase, Thioredoxin Reductase and Thioredoxin Peroxidase-Encoding Genes of *Magnaporthe oryzae* during Rice Blast Disease

**DOI:** 10.1371/journal.pone.0087300

**Published:** 2014-01-24

**Authors:** Jessie Fernandez, Richard A. Wilson

**Affiliations:** Department of Plant Pathology, University of Nebraska-Lincoln, Lincoln, Nebraska, United States of America; University of Wisconsin – Madison, United States of America

## Abstract

Understanding how pathogenic fungi adapt to host plant cells is of major concern to securing global food production. The hemibiotrophic rice blast fungus *Magnaporthe oryzae*, cause of the most serious disease of cultivated rice, colonizes leaf cells asymptomatically as a biotroph for 4–5 days in susceptible rice cultivars before entering its destructive necrotrophic phase. During the biotrophic growth stage, *M. oryzae* remains undetected in the plant while acquiring nutrients and growing cell-to-cell. Which fungal processes facilitate *in planta* growth and development are still being elucidated. Here, we used gene functional analysis to show how components of the NADPH-requiring glutathione and thioredoxin antioxidation systems of *M. oryzae* contribute to disease. Loss of glutathione reductase, thioredoxin reductase and thioredoxin peroxidase-encoding genes resulted in strains severely attenuated in their ability to grow in rice cells and that failed to produce spreading necrotic lesions on the leaf surface. Glutathione reductase, but not thioredoxin reductase or thioredoxin peroxidase, was shown to be required for neutralizing plant generated reactive oxygen species (ROS). The thioredoxin proteins, but not glutathione reductase, were shown to contribute to cell-wall integrity. Furthermore, glutathione and thioredoxin gene expression, under axenic growth conditions, was dependent on both the presence of glucose and the *M. oryzae* sugar/ NADPH sensor Tps1, thereby suggesting how glucose availability, NADPH production and antioxidation might be connected. Taken together, this work identifies components of the fungal glutathione and thioredoxin antioxidation systems as determinants of rice blast disease that act to facilitate biotrophic colonization of host cells by *M. oryzae*.

## Introduction

Fungal diseases of plants represent a categorical defeat of the host innate immune system by the pathogen. Plants rely on two lines of basal defenses [1]–[4] to contain microbial infections: pathogen-associated molecular pattern (PAMP)- triggered immunity (PTI) [1], [2] and a stronger version of PTI called effector-triggered immunity (ETI) [1], [2], [5]. However, pathogens such as the rice blast fungus *Magnaporthe oryzae* [6] can spend at least part of their lifecycle growing undetected in host cells [7], [8]. How *M. oryzae* interferes with plant defenses to initially achieve colonization is only just becoming apparent [9]–[13]. Less clear is how plant defense suppression is integrated with the metabolic demands of the fungus in order to sustain cell-to-cell biotrophic growth in rice cells [14]. Understanding how this might be achieved would enhance our fundamental knowledge of the processes governing rice blast disease.

Rice blast is the most serious disease of cultivated rice, a threat to global food security, and a problem compounded by climate change and modern agricultural practices [6], [14]–[18]. During infection, *M. oryzae* elaborates a specialized structure called an appressorium on the surface of the rice leaf [6], [19], [20]. Penetration of the rice cuticle occurs due to an accumulation of hydrostatic turgor pressure inside the appressorium that acts on a septin-dependent penetration peg emerging at the base of the cell [19]. From the penetration peg, the fungus produces a thin, filamentous primary hypha in the apoplastic space that, in compatible interactions, differentiates into bulbous invasive hyphae (IH) [21]. IH grows within the first infected plant cell surrounded by the plant-derived extra-invasive hyphal membrane (EIHM). At 32 – 36 hour post inoculation (hpi) the fungus develops thin, filamentous IH that move to neighboring cells via plasmodesmata [8], [21], where they differentiate into bulbous IH once more. Successive colonization of living rice cells by IH is accompanied by the secretion of apoplastic and cytoplasmic effector proteins [8], [22] until necrotrophy commences. A compatible interaction between *M. oryzae* and susceptible rice hosts therefore requires overcoming PTI and avoiding ETI to initiate colonization, followed by the prolonged suppression of plant defenses during biotrophic growth.

Plant reactive oxygen species (ROS) production is a feature of PTI and ETI [1], [2], [23] and some *M. oryzae* genes necessary for the neutralization of plant ROS have been characterized [24]. Huang and colleagues [10] identified a gene, *HYR1*, which encodes a glutathione peroxidase and is involved in ROS detoxification. Δ*hyr1* mutants were shown to be unable to tolerate high concentrations of H_2_O_2_ under axenic growth conditions, demonstrated a decreased ability to tolerate ROS generated by a susceptible plant, and were impaired in lesion formation. In another study, a serine-rich protein, known as Defense Suppressor 1 (Des1) was identified from a T-DNA insertional mutant library as having a role in pathogenicity. Des1 was shown to be important for neutralizing host-derived ROS during *M. oryzae* infection and preventing the strong induction of plant defense responses [11]. In contrast to Hyr1 and Des1, the secreted *M. oryzae* catalase CatB was not shown to be important for neutralizing plant-derived ROS at the site of penetration but rather for strengthening cell walls [25], while a secreted catalase-peroxidase, CpxB, is needed for neutralizing plant-derived ROS during early infection but not for pathogenicity [26]. *M. oryzae* also produces endogenous ROS bursts during appressorial formation [27], a process requiring NADPH oxidases and integral to appressorial function [20].

The outcome of plant defense suppression is biotrophic growth of *M. oryzae* in rice cells [14], [18], [21]. An important regulator of *M. oryzae* pathogenicity is the sugar sensor trehalose-6-phosphate synthase 1 (Tps1). In response to the sensing of its substrate glucose 6-phosphate (G6P), Tps1 controls NADPH levels to mediate genetic responses to changing nutrient and redox conditions [14], [28]–[31]. G6P sensing by Tps1 elevates glucose 6-phosphate dehydrogenase (G6PDH) activity to stimulate NADPH production from G6P in the pentose phosphate pathway (PPP). This results, via an NADPH-dependent signaling pathway, in the induced expression of a number of genes, including some encoding NADPH-requiring enzymes [29]. Elevated NADPH levels can displace G6P from the active site of Tps1, presumably preventing Tps1 induction of G6PDH activity and reducing NADPH production. Together, these observations describe a redox homeostatic mechanism whereby on the one hand, NADPH production is balanced with G6P availability by the interactions of these molecules at the Tps1 active site, and on the other hand, the expression of genes encoding NADPH-requiring enzymes is dependent on NADPH availability. Evidence that this NADPH-dependent genetic switch mechanism operates *in planta* comes from the observations that overexpressing *G6PDH* or disrupting the downstream transcription factor inhibitors Nmr1-3 in Δ*tps1* strains at least partially restores their infection capabilities [29]. However, which Tps1-controlled, NADPH-requiring enzymatic activities – if any - are necessary for rice infection is not known.

We wished to address this gap in our knowledge about Tps1 function and sought to determine NADPH-dependent outputs of the Tps1 signaling pathway that impact pathogenicity. Here, we identified the *M. oryzae* glutathione and thioredoxin antioxidation systems as important NADPH-requiring processes, essential for infection, whose gene expression is controlled by Tps1 in response to glucose. Glutathione is a tripeptide antioxidant formed from cysteine, glutamic acid and glycine by the action of glutamate-cysteine ligase and glutathione synthase [32]. In the reduced state, the thiol group of the glutathione cysteine can act as an electron donor to protect against oxidative stress when cells are exposed to ROS, in the process converting reduced glutathione (GSH) to its oxidized form glutathione disulfide (GSSG). GSSG is recycled to GSH by glutathione reductase (Gtr1), a flavoprotein that uses NADPH as an electron donor [32]. GSH is a co-factor for glutaredoxins (small proteins involved in maintaining the redox status of target proteins [32], [33]) and glutathione peroxidase, involved in reducing lipid hydroperoxides [34] and encoded by *HYR1* in *M. oryzae* [10]. Thioredoxins play a similar role to glutaredoxins and are small, ubiquitous NADPH-requiring proteins involved in ROS scavenging [32], [35]. In yeast, electron flow from NADPH forms a redox-sensitive disulfide in thioredoxin, which can reduce the disulfide linkages within thioredoxin peroxidase (Tpx1) [36]. Reduced thioredoxin is regenerated by the action of thioredoxin reductase (Trr1). Using live-cell imaging of infected rice leaf sheaths, we determined that mutant *M. oryzae* strains resulting from the targeted deletion of genes encoding glutathione reductase (*GTR1*), thioredoxin reductase (*TRR1*) or thioredoxin peroxidase (*TPX1*) could penetrate rice cuticles but were impaired in cell-to-cell movement and symptom development. This indicates these NADPH-requiring antioxidation systems are essential for biotrophic growth and fungal virulence. Moreover, the glutathione system was shown to act entirely in the plant during infection – likely in order to neutralize host-derived ROS - because glutathione reductase was not required for appressorial formation or function. In contrast, both Δ*trr1* and Δ*tpx1* mutant strains produced aberrant appressoria on leaf surfaces (but formed normal appressoria on artificial hydrophobic surfaces) and were impaired in plant penetration. This work thus gives new insights into the physiological processes underlying rice infection and illustrates how nutrient availability might be integrated with fungal metabolism, via Tps1, in order to facilitate rapid host cell colonization by the blast fungus.

## Results

### Δ*tps1* strains are sensitive to oxidative stress compared to WT

**Figure 1:**
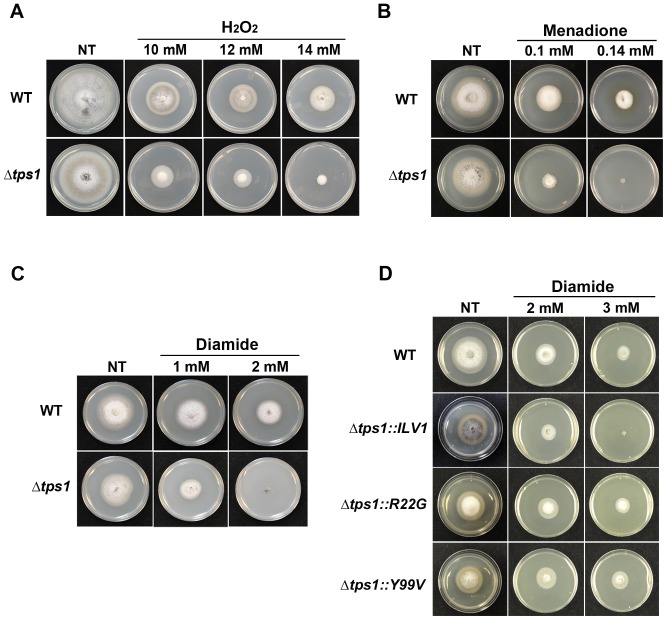
G6P sensing by Tps1 is required for resistance to oxidative stress. WT and Δ*tps1* strains were inoculated as 10 mm mycelial plugs onto 55 mm diameter plates of complete media (CM) containing H_2_O_2_ (**A**), menadione (**B**) or diamide (**C**) at the concentrations indicated. Growth of additional strains was also tested on diamide (**D**), including the new sulphonyl urea resistant Δ*tps1* strain generated in this study (Δ*tps1::ILV1*), and Δ*tps1* strains expressing Tps1 proteins carrying the point-mutations R22G or Y99V in the G6P binding pocket of the active site (Δ*tps1::R22G* and Δ*tps1::Y99V*, respectively). Together, these results suggest G6P sensing, but not Tps1 catalytic activity, is sufficient to restore resistance to oxidative stresses in Δ*tps1* strains. Images were taken after 7 (**A**) and 5 (**B-D**) days growth. NT =  no treatment. Compounds were added at the final concentrations indicated.

The reducing power in cells is determined by NADPH, which serves as an electron donor in reductive biosynthesis, provides the reducing equivalents for neutralizing ROS through the regeneration of cellular antioxidation systems, and is required in the generation of ROS itself [35], [37]. In *M. oryzae,* Tps1 couples NADPH production to the expression of genes encoding putative NADPH-dependent enzymes [29] such that Δ*tps1* strains are defective in both the production of NADPH [28] and the expression of genes encoding enzymes that require NADPH, such as nitrate reductase [29]. Genes residing in clusters that might be involved in the reductive biosynthesis of secondary metabolites also require Tps1 for expression [30]. In this study, we asked if NADPH depletion in Δ*tps1* strains impacts antioxidation and if so, are the NADPH-requiring systems involved relevant to the infection process? Following previously described protocols [10], [11], we grew the ▵*tps1* mutant strain used in our previous studies [28], [29], [38] on solid complete media (CM) containing H_2_O_2_, the free radical generator menadione, or the glutathione-specific thiol oxidant diamide, and compared its growth to the wild type (WT) isolate Guy11. We found that the ▵*tps1* mutant strain was sensitive to all three oxidants compared to WT (Figure 1A-C).

To confirm that sensitivity to diamide and other oxidative stresses was solely due to the loss of Tps1 function, we sought to determine if resistance to oxidative stress could be restored by complementing the Δ*tps1* mutant strain with functional copies of the *TPS1* gene, and also if sensitivity to oxidative stresses could be recapitulated in independently generated Δ*tps1* strains. Previous studies [28], [31] have characterized Δ*tps1* strains carrying copies of the *TPS1* gene encoding amino acid point mutations in the active site of the enzyme. Two changes, R22G and Y99V, generated proteins that allowed the G6P substrate into the active site but were catalytically inactive and unable to produce the trehalose intermediate trehalose-6-phosphate (T6P) [28]. When constructs encoding these Tps1 variants were introduced into Δ*tps1* strains, the resulting Δ*tps1::R22G* and Δ*tps1::Y99V* strains could not produce trehalose [28] but were restored for pathogenicity and carbon catabolite repression [28], [31]. These results indicated that the signaling function of Tps1 occurs in response to G6P sensing at the active site independently of Tps1 biosynthetic capabilities [28]. Figure 1D shows that the Δ*tps1::R22G* and Δ*tps1::Y99V* strains were restored for resistance to diamide, indicating G6P sensing by Tps1 is necessary and sufficient to restore oxidative stress responses in Δ*tps1* strains. In addition, the Δ*tps1* mutant strain used in Figure 1A-C was generated by homologous replacement of *TPS1* by *Hph* conferring hygromycin resistance [39]. To confirm that increased sensitivity to oxidative stress was a general feature of impaired Tps1 function, we generated an independent Δ*tps1* strain using our split-marker, high throughput gene deletion strategy [29] by replacing the *TPS1* coding region with *ILV1* conferring resistance to sulphonyl urea. Figure S1A shows that the resulting sulphonyl urea resistant Δ*tps1* strain, hereby designated Δ*tps1::ILV1*, was unable to utilize nitrate as a sole nitrogen source, a hallmark of the original hygromycin-resistant Δ*tps1* strain (hereby designated Δ*tps1*) [28], [31]. The Δ*tps1::ILV1* strain was also, like Δ*tps1*, sensitive to diamide (Figure 1D) and menadione (Figure S1B). Taken together, we conclude that *M. oryzae* antioxidation defense requires G6P sensing by Tps1.

### The *M. oryzae* glutathione antioxidation system is required *in planta*

Diamide is a thiol-oxidizing agent that mediates the direct oxidation of reduced glutathione (GSH) to GSSG [40]. In the yeast *Saccharomyces cerevisiae*, mutants of the glutathione antioxidation system cannot recycle GSSG to GSH and are sensitive to diamide [41]. Sensitivity of *M. oryzae* Δ*tps1* strains to diamide might indicate the NADPH-requiring glutathione antioxidation system is under Tps1 control in response to G6P sensing. To determine if glutathione metabolism is an important output of the Tps1 signaling pathway, we next sought to describe the role of glutathione antioxidation in rice blast disease.

**Figure 2:**
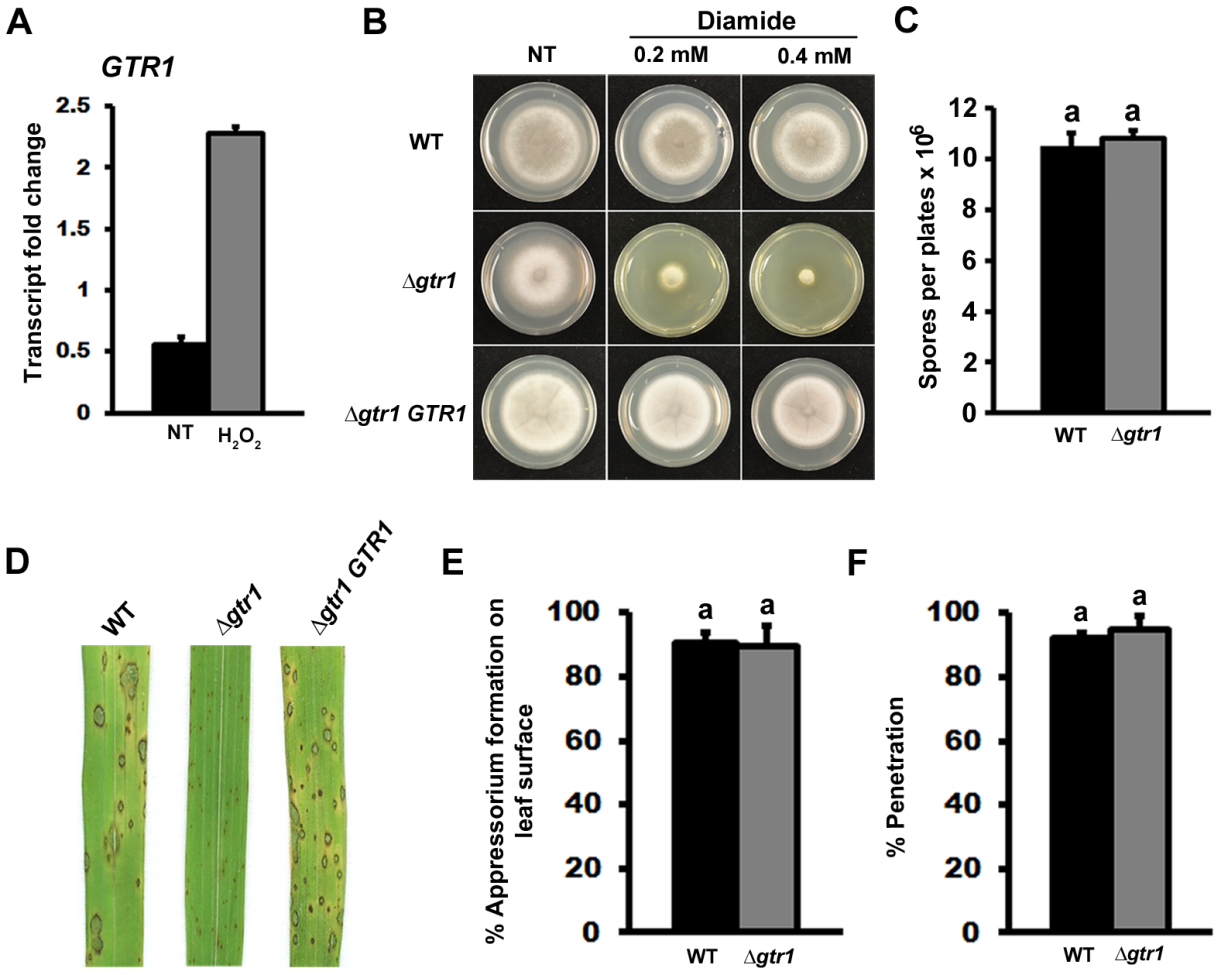
*GTR1* is required for pathogenicity. (**A**) Expression of the glutathione reductase-encoding gene *GTR1* in WT strains was induced more than two-fold in the presence of 5 mM H_2_O_2_ compared to CM alone. NT = no treatment. Results were normalized against the expression of the β-tubulin gene *TUB2*. (**B**) Disruption of the *GTR1* coding region resulted in sensitivity of Δ*gtr1* strains to diamide compared to WT strains. Images were taken after 5 days growth. NT =  no treatment. (**C**) ▵*gtr1* strains were not affected in conidiation on CM media compared to parental WT strains. (**D**) Δ*gtr1* strains were reduced in virulence compared to WT parental strains when applied to leaves of the susceptible rice cultivar CO-39. (**E**) The rates of appressorium formation by Δ*gtr1* strains on rice leaf surfaces were not significantly different (*Student’s t-test* p≤0.05) to those observed for WT. (**F**) Appressoria of WT and Δ*gtr1* strains had the same rates of leaf cuticle penetration at 36 hpi. (**C, E, F**) Values are the mean of at least three independent replicates. Error bars denote SD. Bars with the same letters are not significantly different (*Student’s t-test* p≤0.05).

A putative glutathione reductase-encoding gene, locus number MGG_08895 (which we have called *GTR1*) was identified by sequence homology in the *M. oryzae* genome database [42]. Figure 2A shows, using quantitative real time PCR (qPCR), that *GTR1* expression in WT was induced more than two-fold in the presence of H_2_O_2_. To investigate the function of *GTR1*, we used targeted gene replacement to generate ▵*gtr1* deletion strains. Compared to WT, *Δgtr1* strains were sensitive to H_2_O_2_ (Figure S2A), menadione (Figure S2B) and the thiol oxidizing agent diamide (Figure 2B), confirming a role for Gtr1 in antioxidation and survival under oxidative stress conditions. Sensitivity was reversed in Δ*gtr1* strains by re-introducing a functioning copy of the *GTR1* gene (Figure 2B).

Under normal plate growth conditions, Δ*gtr1* strains could sporulate at wild type rates (Figure 2C; *Student’s t-test* p  =  0.37). However, unlike WT or Δ*gtr1 GTR1* complementation strains, Δ*gtr1* strains were greatly reduced for pathogenicity when spores were applied to whole rice leaves (Figure 2D). Reduced virulence was not due to perturbed appressorial function because Δ*gtr1* strains could form appressoria on rice leaf surfaces at the same rate as WT (Figure 2E; *Student’s t-test* p  =  0.76) and with the same rates of cuticle penetration (Figure 2F; *Student’s t-test* p  =  0.37).

**Figure 3:**
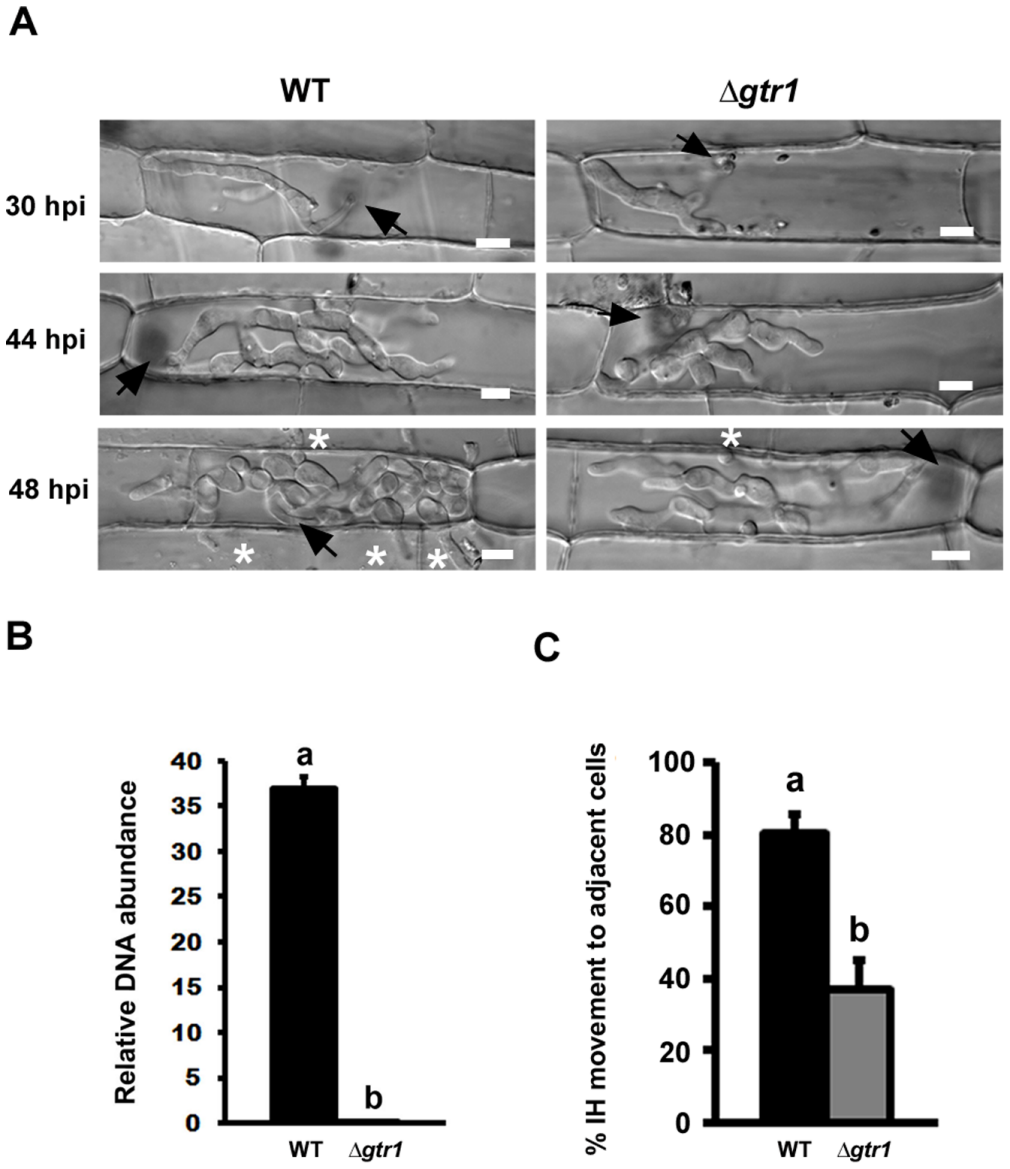
*GTR1* is required for *in planta* growth. (**A**) Live-cell imaging demonstrated Δ*gtr1* strains were reduced for *in planta* growth on detached rice leaf sheaths compared to WT strains. Arrows indicate appressoria on the surface of the leaf corresponding to the point of penetration. Asterisks indicate spread of hyphae to adjacent cells. Scale bar is 5 µm. (**B**) At 72 hpi, leaves infected with WT were found to contain 37-fold more fungal DNA than leaves infected with *Δgtr1* strains. DNA was isolated from infected rice leaves and fungal DNA abundance was determined using primers specific for *MoACT1* and normalized against the rice actin gene. (**C**) IH growth to adjacent cells was significantly reduced (Student’s t-test p≤0.05) in ▵*gtr1* strains compared to WT at 48 hpi. (**B** and **C**) Values are the mean of at least three independent replicates. Error bars denote SD. Bars with the same letters are not significantly different (*Student’s t-test* p≤0.05).

We undertook live-cell imaging using detached rice leaf sheaths to observe how Δ*gtr1* IH developed within host cells. We found that Δ*gtr1* strains could elaborate IH from primary hyphae, but the growth of ▵*gtr1* strains was delayed *in planta* compared to WT (Figure 3A). Three time points are shown to illustrate how Δ*gtr1* strains grew less extensively than WT in host cells. This delay in growth was quantified to show that at 72 hpi, leaves infected with Δ*gtr1* strains contained 37 ± 5 - fold (n = 3) less fungal DNA than those infected with WT (Figure 3B). Moreover, a significant reduction was observed in the mean movement of Δ*gtr1* IH into adjacent cells at 48 hpi compared to WT (*Student’s t-test* p  =  0.0014; Figure 3C).

Overall, our data suggests *GTR1* contributes significantly to IH growth in rice cells and full symptom development but is not required for appressorial formation or function. Therefore, the role of *GTR1* in fungal virulence appears to lie solely in the plant.

### The thioredoxin system is required for infection-related development and host colonization

**Figure 4:**
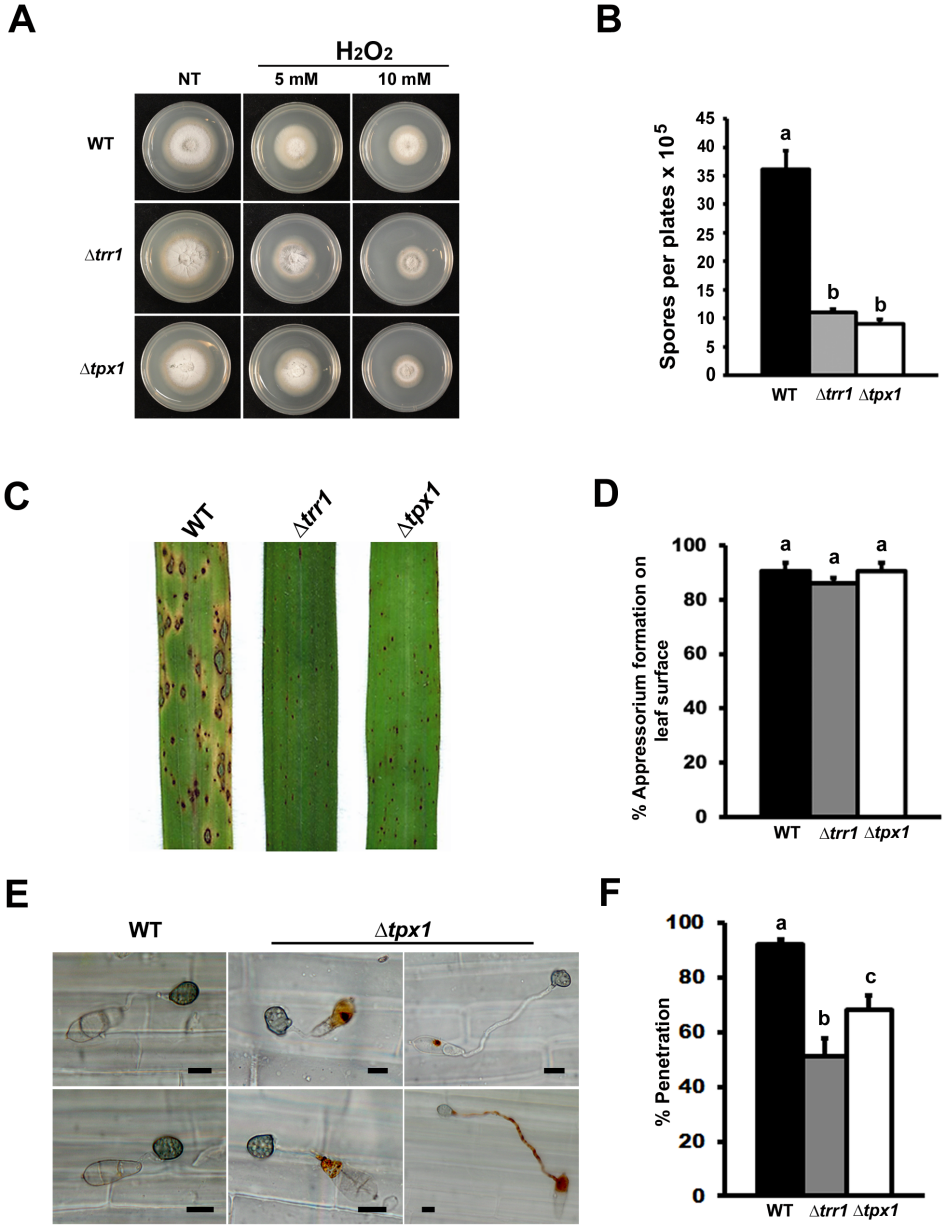
The thioredoxin antioxidation system is essential for appressorium function and pathogenicity. **(A)** Δ*trr1* and Δ*tpx1* thioredoxin mutant strains were sensitive to H_2_O_2_ compared to WT on CM at the concentrations shown. NT  =  no treatment. (**B**) ▵*trr1* and Δ*tpx1* mutant strains were significantly reduced (*Student’s t-test* p≤0.05) in conidiation on CM media compared to parental WT strains. (**C**) Δ*trr1* and Δ*tpx1* mutant strains were reduced in virulence compared to WT parental strains when applied to leaves of the susceptible rice cultivar CO-39. (**D**) Rates of appressorial formation on rice leaf surfaces were not significantly different (*Student’s t-test* p≤0.05) for Δ*trr1* strains or Δ*tpx1* strains compared to WT. (**E**) Δ*tpx1* strains produced a distinctive pigment in the conidium and/ or germ tube when in contact with the leaf surface that was not seen for WT strains. Scale bars: 10 µm. (**F**) Appressorial penetration rates of leaf cuticles were significantly reduced (*Student’s t-test* p≤0.05) in ▵*trr1* and ▵*tpx1* strains compared to WT. (**B, D, F**) Values are the mean of three independent replicates. Error bars are SD. Bars with the same letter are not significantly different (*Student’s t-test* p≤0.05)**.**

**Figure 5:**
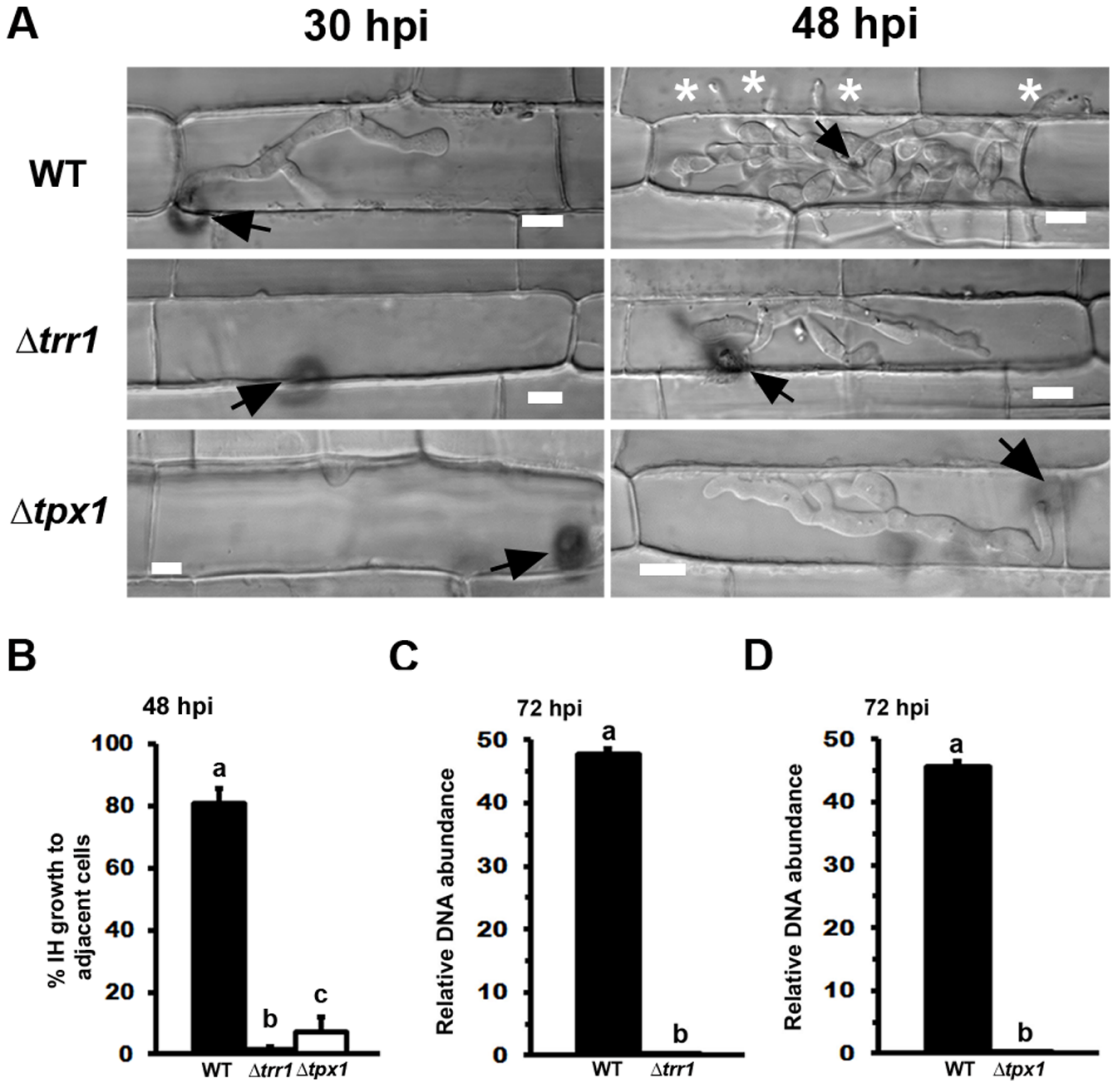
Mutants of the thioredoxin antioxidation system are impaired in biotrophic growth and cell-to-cell movement in the host. (**A**) Live-cell imaging at 30 hpi and 48 hpi shows *▵trr1* and *▵tpx1* strains were considerably impaired in IH growth in susceptible CO-39 rice leaf sheaths compared to WT. Arrows indicate appressoria on the surface of the leaf corresponding to the point of penetration. Asterisks indicate hyphal movement to adjacent rice cells. Scale bar is 5 µm. (**B**) IH growth to adjacent cells of *▵trr1* and *▵tpx1* strains was significantly (Student’s t-test p≤0.05) impaired compared to WT strains at 48 hpi. Values are the mean of three independent replicates. Error bars denote SD. Bars with the same letters are not significantly different (Student’s t-test p≤0.05). The relative amount of fungal DNA (a proxy for relative fungal mass) from leaves infected with strains of Δ*trr1* (**C**) and Δ*tpx1* (**D**) was 45- and 47-fold less, respectively, compared to WT. Values are the mean of three independent replicates. Error bars denote SD. Bars with the same letters are not significantly different (Student’s t-test p≤0.05).

Exploring the Δ*tps1* phenotype had revealed glutathione antioxidation as a potentially important NADPH-dependent infection process, and subsequent characterization of Δ*gtr1* strains confirmed this system is required for the *in planta* colonization of rice. Glutathione acts to reduce glutaredoxins, small redox enzymes that share many of the cellular functions of thioredoxin [43], [44]. We next asked if - and how - the thioredoxin antioxidation system might also contribute to rice infection by *M. oryzae*. We identified putative thioredoxin reductase (*TRR1*; MGG_ 01284) and thioredoxin peroxidase (*TPX1;* MGG_07503) orthologues in the *M. oryzae* genome database [42] and generated ▵*trr1* and ▵*tpx1* deletion strains that were significantly (*Student’s t-test* p≤0.05) more sensitive to 10 mM H_2_O_2_ (Figure 4A and Figure S3), but not diamide or menadione (data not shown), than WT. Figure 4B shows that ▵*trr1* and ▵*tpx1* strains were significantly reduced in conidiation compared to WT strains on CM (*Student’s t-test* p  =  0.0001). When inoculated onto susceptible rice plants, ▵*trr1* and ▵*tpx1* strains were unable to produce expanded necrotic lesions compared to WT (Figure 4C). ▵*trr1* and ▵*tpx1* strains were not defective in their rates of appressorium formation on rice leaf surfaces compared to WT (Figure 4D) or on artificial hydrophobic surfaces (not shown). However, although ▵*trr1* and ▵*tpx1* strains exhibited normal radial growth on CM (Figure S4) and produced normal-looking appressoria on artificial hydrophobic surfaces (Figure S4), both ▵*trr1* and ▵*txp1* strains produced odd-shaped appressoria on rice leaf surfaces (Figure S4). Moreover, ▵*tpx1* strains, but not Δ*trr1*, produced a distinctive pigment when forming appressoria on rice leaf surfaces but not hydrophobic surfaces (Figure 4E and Figure S4). Both ▵*trr1* and ▵*tpx1* strains formed appressoria that were significantly defective (*Student’s t-test* p =  0.0005 and 0.0018, respectively) in penetrating rice cuticles compared to WT (Figure 4F). Following penetration, we also observed severe delays in the rate of rice cell colonization by ▵*trr1* and ▵*tpx1* strains (Figure 5A). In both cases, IH movement to cells adjacent to the point of infection was significantly attenuated compared to WT (Figure 5B). To quantify the observed reduction in virulence, the relative amount of fungal DNA in rice cells infected with *▵trr1* (Figure 5C) and *▵tpx1* (Figure 5D) strains was determined at 72 hpi and compared to WT. Both Δ*trr1* and Δ*tpx1* strains were significantly reduced for fungal DNA content in host tissues compared to WT at this timepoint.

Taken together, genes of the *M. oryzae* thioredoxin antioxidation system are required for sporulation and appressorial development, penetration, cell-to-cell movement and full symptom development on rice leaves.

### The glutathione, but not thioredoxin, antioxidation system suppresses host ROS accumulation

**Figure 6:**
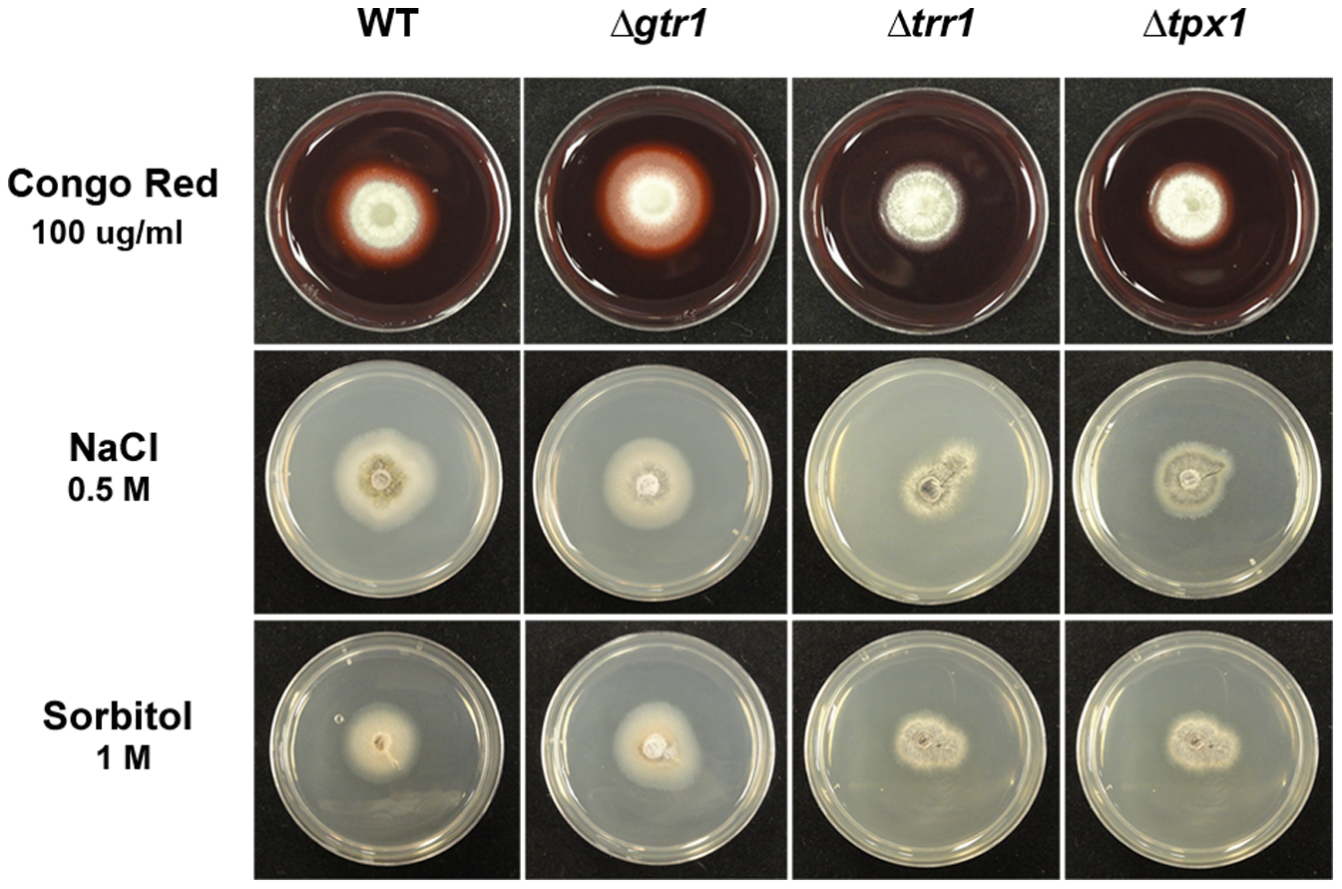
Thioredoxin mutant strains Δ*trr1* and Δ*tpx1* are sensitive to the cell wall disrupter Congo Red and the osmolytes NaCl and sorbitol. Stressors were added to CM at the concentrations shown. Images were taken after 5 days growth.

**Figure 7:**
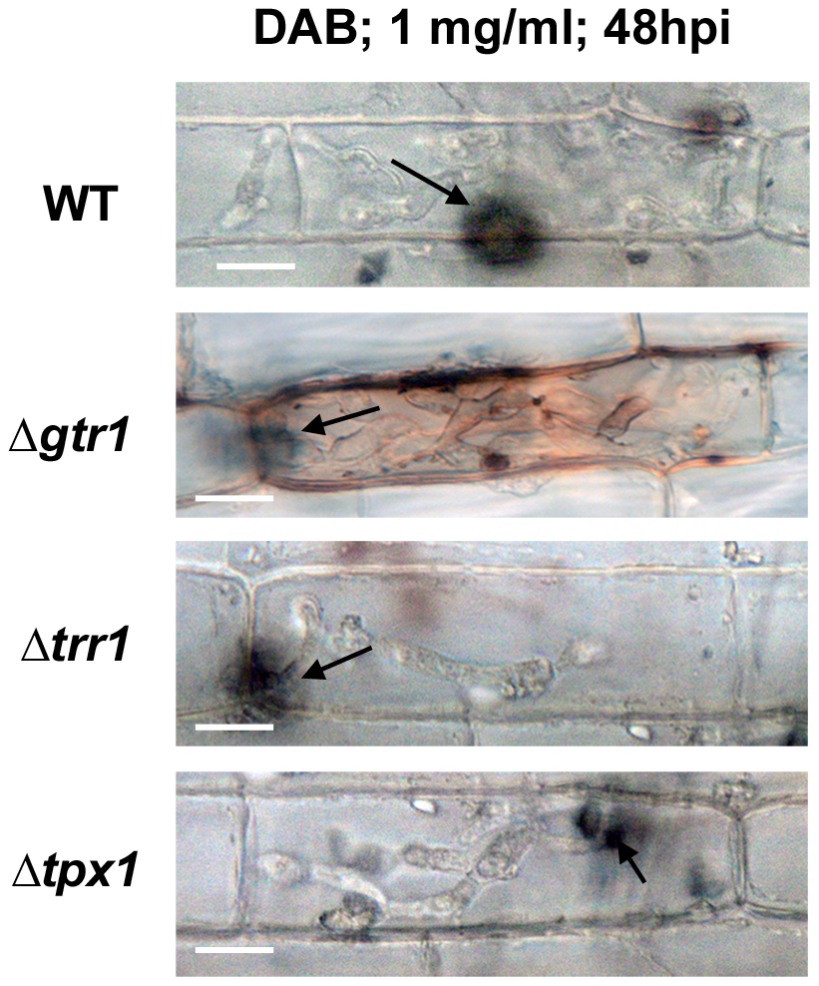
*GTR1* but not *TRR1* or *TPX1* is required for suppressing host oxidative defenses. Detached rice leaf sheaths were inoculated with the indicated strains, stained with DAB at 48 hpi, and observed under the light microscope. Only cells infected with Δ*gtr1* strains showed significant DAB staining, resulting in orange pigment formation and indicative of H_2_O_2_ accumulation at the penetration site. Bar is 5 µm. Arrow indicates the appressorial point of penetration.

The above results suggest that although genes of both the glutathione and thioredoxin antioxidation systems are required for full growth *in planta*, they are not equivalent in their roles regarding sporulation or appressorial development and function. Moreover, ▵*trr1* and ▵*tpx1* deletion strains were more sensitive to the cell-wall assembly inhibitor Congo Red, and osmotic stresses, than WT or Δ*gtr1* strains (Figure 6). Non-equivalency of function extends to the host interior, where Figure 7 shows that rice cells infected with Δ*gtr1* strains stained strongly when incubated with 3,3’-diaminobenzidine (DAB) - indicating the accumulation of hydrogen peroxide (H_2_O_2_) at infection sites [10], [11] - compared to WT, which was not stained. In contrast to Δ*gtr1*, rice cells infected with Δ*trr1* and Δ*tpx1* strains stained much less strongly with DAB even though IH growth was more severely attenuated in these strains. This suggests *GTR1* is involved in ameliorating extracellular plant host oxidative defenses, but the thioredoxin antioxidation system plays less of a role in neutralizing plant ROS during early infection.

### Tps1 and glucose availability control glutathione and thioredoxin gene expression in axenic shake cultures

**Figure 8:**
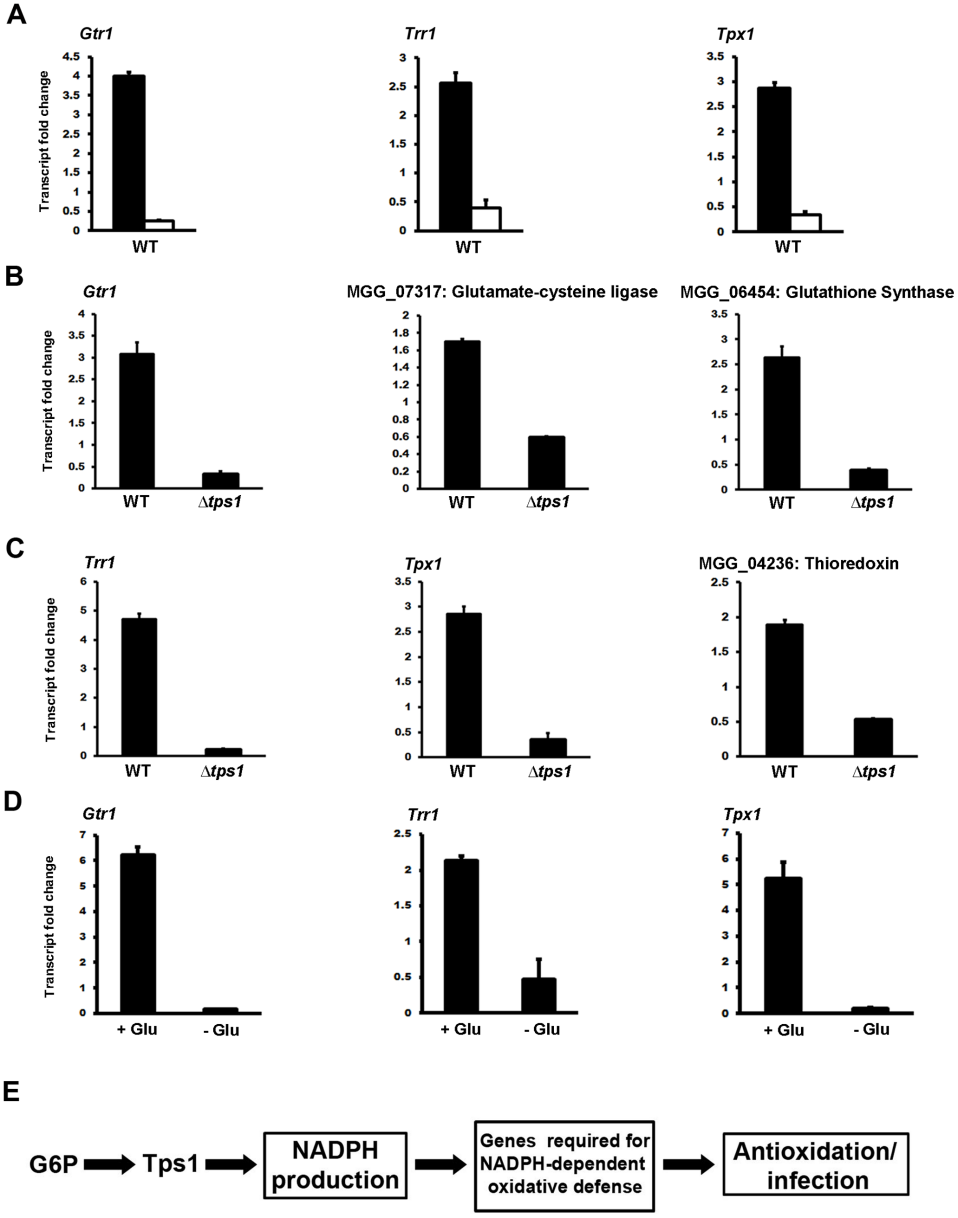
Tps1 controls glutathione and thioredoxin gene expression in response to glucose. **(A)** In WT, *GTR1*, *TRR1* and *TPX1* expression was induced on 1% (w/v) glucose minimal media (GMM) with nitrate as a sole nitrogen source (ie. NADPH-replete conditions, closed bars) compared to GMM with ammonium as a sole nitrogen source (open bars). (**B**) Tps1 controls the expression of glutathione and (**C**) thioredoxin antioxidation genes during growth on GMM with nitrate. The expression of genes involved in glutathione recycling (*GTR1*) and glutathione biosynthesis (MGG_07317 and MGG_06454) was downregulated in Δ*tps1* compared to WT strains following growth on GMM with nitrate. Genes involved in thioredoxin biosynthesis (MGG_04236), recycling (*TRR1*) and use (*TPX1*) were also downregulated in Δ*tps1* compared to WT strains on GMM with nitrate. (**D**) The antioxidation genes characterized in this report are glucose-responsive. Expression levels for *GTR1*, *TRR1* and *TPX1* were determined following the growth of WT on nitrate-containing minimal media with (+G) or without (-G) 1% (w/v) glucose. (**A-D**) Values are the mean of three independent technical replicates and at least two biological replicates. Error bars are SD. Gene expression results were normalized against the expression of the ß-tubulin gene (*TUB2*). Results represent fold changes in gene expression when comparing two strains (**B, C**) or two growth conditions (**A, D**). (**E**) Model summarizing the role of Tps1 in antioxidation in response to G6P sensing.

Sensitivity of Δ*tps1* strains to diamide and other oxidative stresses indicated G6P sensing by Tps1 was required for antioxidation defenses. Subsequent functional characterizations confirmed the importance of the NADPH-dependent glutathione and thioredoxin antioxidation systems to rice infection. We next sought to connect these observations by confirming the NADPH-dependent regulation of glutathione and thioredoxin antioxidation gene expression by Tps1 in response to G6P sensing. In previous reports, we had shown how NADPH-requiring genes were induced under Tps1-dependent, NADPH-replete conditions [29], [31]. In WT, growth on nitrate as a sole nitrogen source induces NADPH production via Tps1 control of G6PDH in response to G6P sensing. NADPH provides the reducing power for nitrate reductase to metabolize nitrate to nitrite, which is further reduced to ammonium by nitrite reductase [28]. Consequently, increased NADPH production is not observed during the growth of WT on ammonium media, nor by Δ*tps1* strains on nitrate media [28]. Moreover, WT growth on nitrate media induces the expression of other genes encoding NADPH-requiring enzymes in addition to nitrate reductase via the NADPH-dependent genetic switch described in [29]. This induction is abolished in Δ*tps1* strains. Consistent with the NADPH-dependent switch model [29], qPCR analysis showed *GTR1*, *TRR1* and *TPX1* gene expression was induced in WT following growth in nitrate media (ie. NADPH-replete conditions) compared to ammonium media (Figure 8A). In addition, the induction of *GTR1*, *TRR1* and *TPX1* gene expression – in addition to the expression of genes encoding glutamate-cysteine ligase, glutathione synthase and thioredoxin itself - was found to be Tps1-dependent on nitrate media (Figure 8B and 8C). Furthermore, *GTR1*, *TRR1* and *TPX1* gene expression in WT was abolished in the absence of glucose on nitrate media (Figure 8D). Taken together, Figure 8A-D shows how genes of the glutathione and thioredoxin antioxidation systems are expressed in a glucose- and Tps1-dependent manner under NADPH-replete conditions. Combined with the growth data in Figure 1D, this suggests Tps1 - via G6P sensing and gene regulation - might coordinate glucose availability in the fungal cell with the production of NADPH in order to fuel at least some antioxidation systems during rice infection (Figure 8E).

## Discussion

The biotrophic stage of *M. oryzae* rice infection involves a complex molecular interplay between host cell and pathogen [8], [22], but many of the processes involved and, in particular, their regulation, remain to be elucidated. Here, we have revealed how components of the glutathione and thioredoxin antioxidation systems are essential for *in planta* colonization and symptom development. Moreover, we have shown that glutathione and thioredoxin genes are expressed in a glucose- and Tps1-dependent manner, thus suggesting how NADPH-requiring antioxidation might be regulated and fuelled as the fungus exploits available sources of glucose during infection (Figure 8E).

This study used plate testing as a tool to identify glutathione reductase as a Tps1-dependent, NADPH-requiring antioxidation process important for rice infection. Sensitivity of Δ*tps1* strains to diamide indicated Tps1 might control the glutathione antioxidation system (Figure 1). To determine the role of glutathione in host infection, Δ*gtr1* strains were generated using targeted gene deletion of the *M. oryzae GTR1* orthologue. Δ*gtr1* strains demonstrated increased sensitivity to oxidative stresses during axenic growth compared to WT (Figure 2B) and were restricted in their ability to colonize host tissue relative to WT (Figure 3) despite producing functional appressoria on leaf surfaces (Figure 2E). DAB staining of infected leaf sheaths demonstrated a reduced capacity of Δ*gtr1* strains to suppress plant ROS compared to WT (Figure 7). Because spore production, appressorium formation and appressorium function was not affected in Δ*gtr1* strains (Figure 2), the significance of these findings lie in suggesting that the NADPH-dependent regeneration of the glutathione antioxidation system is an *in planta*-specific process essential for host tissue colonization. Such a stage-specific role for glutathione reductase has, to our knowledge, not been described before in pathogenic fungi and extends our knowledge of this important enzyme.

In contrast to the apparent *in planta*-specific role of glutathione reductase, loss of components of the thioredoxin antioxidation system affected many aspects of the fungal life cycle. Δ*trr1* and Δ*tpx1* thioredoxin mutants were more sensitive to 10 mM H_2_O_2_ than WT (Figure 4A and Figure S3). Δ*trr1* and Δ*tpx1* strains produced appressoria with the same frequency as WT on rice leaves (Figure 4D), but they were aberrant in shape (Figure S4) and deficient in function (Figure 4F). Moreover, Δ*tpx1* strains produced a striking pigment in germ-tubes and appressoria (Figure 4E), but only in the presence of the host leaf. On artificial hydrophobic surfaces, both Δ*trr1* and Δ*tpx1* strains produced appressoria indistinguishable from wild type (Figure S2), suggesting a role for thioredoxin antioxidation (but not glutathione) in mediating the *M. oryzae*- rice interaction prior to host invasion. Once inside rice cells, the thioredoxin mutants, like Δ*gtr1* strains, were reduced in cell-to-cell movement (Figure 5). However, unlike Δ*gtr1* strains, rice cells infected with the thioredoxin mutants did not stain with DAB (Figure 7), suggesting this system does not significantly participate in neutralizing host ROS defenses during the early infection of rice epidermal cells. Rather, the importance of *TRR1* and *TPX1* might instead lie in maintaining internal redox balance and/ or functioning in other physiological processes such as cell-wall integrity (as suggested by the sensitivity of Δ*trr1* and Δ*tpx1* strains to Congo Red (Figure 6)). In support of this endogenous metabolic role, we note that Δ*trr1* and Δ*tpx1* deletion strains, but not Δ*gtr1*, demonstrated physiological defects, such as reduced sporulation, in the absence of the host plant, suggesting they are required for normal *ex planta* growth and developmental processes in *M. oryzae*.

Thioredoxin, thioredoxin reductase and thioredoxin peroxidase work together to mitigate oxidative stress [36]. In addition, thioredoxins can contribute to a number of cellular processes such as ribonucleotide reduction and the Calvin cycle by targeting proteins other than thioredoxin peroxidases [43], [45]. It is interesting, therefore, to note that while Δ*trr1* and Δ*tpx1* strains are both reduced for leaf sheath penetration rates compared to WT (Figure 4F), Δ*trr1* strains penetrate rice leaf surfaces with significantly less frequency than Δ*tpx1* strains (Figure 4F). This could indicate that thioredoxin targets proteins in addition to Tpx1 in order to facilitate appressorial penetration. Determining the identity of those additional targets would likely contribute to our understanding of appressorial function.

The wide range of physiological processes affected in the thioredoxin mutants described here are consistent with findings in plants, where plant thioredoxins are shown to have extensive roles in development, growth and cell-to-cell communication [46]. In contrast, the phenotypes of the *M. oryzae* glutathione and thioredoxin mutants are distinct from those of the catalase-defective Δ*catB* strain [25], which - unlike Δ*gtr1* Δ*trr1* and Δ*tpx1* strains - was more resistant to oxidative stress.

Taken together, our work indicates that the glutathione and thioredoxin antioxidation systems have mostly non-equivalent roles in fungal physiology but both are pathogenicity determinants necessary for promoting the biotrophic growth of *M. oryzae* in rice cells, thus making them attractive targets for inhibiting rice blast disease.

The *M. oryzae* glutathione and thioredoxin antioxidation genes were expressed in a glucose-dependent manner (Figure 8D). This is important because both antioxidation processes are fuelled by the reducing power of NADPH. Although NADPH can be produced at different locations in the cell by several enzymes that do not require glucose as a substrate (such as the NAD kinases [35], [37]), our previous work has suggested that G6P flux through the PPP is the major source of NADPH with relevance to blast disease [28], [29]. Indeed, inducing PPP activity in NADPH-defective Δ*tps1* strains by overexpressing *G6PDH* partially restores virulence to this non-pathogenic mutant strain [29]. Furthermore, glucose is shown here to induce antioxidation gene expression through Tps1 (Figure 8B and 8C). Tps1 is required, in response to G6P sensing, for expressing genes encoding NADPH-dependent enzymes and repressing genes required for alternative carbon source utilization [29], [31]. Our results therefore suggest - to our knowledge, for the first time - a genetic connection between G6P and antioxidation that is mediated by Tps1-dependent NADPH production in the PPP (Figure 8E). Moreover, Tps1 control of NADPH-dependent glutathione and thioredoxin antioxidation gene expression in response to G6P provides additional support for the postulated role of Tps1 in maintaining redox homeostasis during pathogenesis [29].

How does the work describe here contribute to a mechanistic understanding of rice blast disease? Recently, we have shown how biotrophic growth is dependent on endogenous sources of purines [47] and methionine [38] despite their abundance in host cells [47], thus experimentally confirming the host environment to be nitrogen-poor during early infection, at least from the perspective of the fungus [18], [47]. We have suggested that this demand for endogenously produced nitrogenous compounds could be a trade-off between the nitrogen requirements of the fungus on the one hand, and the need to maintain the EIHM - a likely barrier to amino acid and purine uptake - on the other [14], [47]. Such a trade-off might persist if *M. oryzae* biotrophic hyphae, similar to those of *Colletotrichum* species [48], function primarily as platforms for effector accumulation and delivery [47]. Indeed, the accumulation of secreted apoplastic effectors between the fungal cell wall and the EIHM supports the use of this strategy - and the need for the EIHM - during rice blast disease [12], [22]. In contrast to nitrogen, sources of glucose might readily cross the EIHM for uptake by IH [47] due to the role of G6P sensing by Tps1 - and NADPH production - in driving infection [29]. The work presented here supports and extends this model of *M. oryzae* biotrophic growth by firstly indicating how Tps1-dependent NADPH production, in response to G6P, might both control and fuel antioxidation in order to permit rapid growth in rice cells (Figure 8E). Secondly, our work, when taken together, suggests *M. oryzae* biotrophic hyphae might function in both effector delivery and glucose-dependent antioxidation, but not nutrient acquisition *per se*, during early infection. Finally, our results fit the testable hypothesis that *M. oryzae* is adapted to thrive in a carbon-rich, nitrogen-poor environment and thus provides a theoretical framework for conceptualizing future discoveries about the response of *M. oryzae* to the host cell milieu.

## Conclusion

In conclusion, the work presented here suggests *M. oryzae* NADPH-dependent antioxidation is important for neutralizing plant-derived ROS and maintaining redox homeostasis and thus gives fresh insights into the metabolic demands of biotrophy. Committing glucose metabolism to the production of NADPH for antioxidation has been observed in other rapidly proliferating entities such as cancer cells [49] and activated macrophages [50] in order to maintain internal redox balance. Such shared metabolic strategies between cell types and across taxa indicate a fundamental requirement for NADPH-dependent antioxidation during rapid growth and/ or host colonization. Understanding how we can perturb *M. oryzae* redox balance during rice infection could thus lead to novel plant protection strategies and impact our understanding of those areas of biology where redox balance and antioxidation are critical. Moreover, determining to what extent glucose metabolism and redox balancing is important for other plant pathogen-host interactions, or for interactions between plants and beneficial mycorrhizal fungi, would be a fascinating future endeavor.

## Materials and Methods

### Fungal isolates, culture conditions and physiological analyses

Guy11 was used as the wild type (WT) isolate for these studies [31] and all mutant strains mentioned in this study were generated from the WT parental strain (Table S1). Standard procedures for the culture and storage of *M. oryzae* were used, as described in [47]. Strains were maintained on complete medium (CM), as described previously [38]. 85 mm diameter plates (unless otherwise stated) were incubated at 24°C under 12 hrs light/dark cycles. Plate images were taken with a Sony Cyber-shot digital camera, 14.1 mega pixels, after 10 days of growth (unless otherwise stated). For sporulation rates, strains were grown on at least three independent CM plates. After 12 days of growth, the spores were harvested and counted using a hemocytometer (Corning). Appressorial development assays were performed, as described previously [38], on hydrophobic microscope coverslips (Fisherbrand). Average values were determined from 50 spores, performed in triplicate [38].

### Fungal transformations and targeted gene deletions

Transformations were performed as described previously [29]. All gene deletion strains were generated using the PCR-based split marker approach (described in [29]) in which the *ILV1* gene, conferring resistance to sulphonyl urea, replaced the native *TPS1, GTR1*, *TRR1* or *TXP1* gene in the WT genome. The PCR primers used are shown in Table S2, and the thermocycler conditions for the first round were 1 min at 95°C initial denaturation, followed by 34 cycles of 95°C for 30 sec denaturation, 63°C for 30 sec annealing and 68°C for 1 min extension. Thermocycler conditions for the second round were the same except for a 3 min extension time. Strains carrying homologous gene replacement of the gene of interest were identified by PCR as described by Wilson et al. [29] using the F1 and R1 oligonucleotide primers shown in Table S2.

### Complementation studies

Full-length copies of each gene were re-introduced into gene deletion strains using the yeast GAP-repair approach described by Zhou et al. [51]. A full-length copy of the each gene, including its native promoter, was amplified using the primers shown in Table S2. Competent cell production and transformation of the XK1-25 strain was performed using the Alkali-cation yeast transformation kit (MP Biomedicals).

### Rice blast pathogenicity assays

Rice blast pathogenicity assays were performed as described previously [29], [31] using three to four week old rice seedlings from the susceptible cultivar, CO-39. Spores were applied as suspensions in a 0.2% gelatin (Difco) solution at a rate of 1×10^5^ spores ml^−1^. Plants were placed in a growth chamber with 12hr light/dark periods. The infected leaves were collected at 5 days and images were taken using an Epson Workforce scanner at a resolution of 300 dpi.

### Rice Leaf Sheath Assay

To visualize fungal colonization of rice epidermal cells, rice leaf sheaths from the susceptible cultivar CO-39 were inoculated with fungal spores (1×10^5^ spores ml^−1^ in 0.20% gelatin) in the hollow interior of the sheaths as described previously [38], [47]. Infected sheaths were observed under a light microscope (Zeiss AxioSkop). Appressorium formation and penetration rates were determined in triplicate as previously described [38], [47]. Mean IH growth rates and movement to adjacent cells, at 48 hpi, was determined from fifty appressoria per treatment, repeated in triplicate, as previously described [38], [47]. Images were taken using a Nikon A1 laser scanning confocal mounted on a Nikon 90i compound microscope at the University of Nebraska-Lincoln Microscopy Center.

### Oxidative, osmotic and cell wall stress conditions

To examine the effect of growth under oxidative stress conditions, WT and mutant strains were grown on CM plates containing the oxidants hydrogen peroxide (H_2_O_2_, 30% in water; Fisher), 2-methyl-1,4-naphthoquinone (menadione; Acros Organics) or diamide [azodicarboxylic acid bis (N,N-dimethylamide) (Sigma)] solutions, at the concentrations shown, following Huang and colleagues [10]. Pictures were taken at 5 days post treatment.

To observe the accumulation of H_2_O_2_ at infection sites, infected rice leaf sheaths (48 hpi) were stained with 3, 3’-diaminobenzidine (DAB, Sigma) as described previously by Chi et al. [11]. Rice sheaths were incubated in 1 mg/ml DAB solution in the dark at room temperature for 8 hours and destained with ethanol: acetic acid solution (94:4 v/v) for 1 hr. Samples were excised and observed under a light microscope.

Mutant strains were tested for cell wall and osmotic stress conditions. Cell wall stress condition was performed by adding 100 ug/ml of Congo Red (Sigma) to solid complete media (CM). Osmotic stress conditions were applied using 0.5 M NaCl (Sigma) and 1 M Sorbitol (Sigma) in CM. Pictures were taken at 5 days post inoculation.

### Gene transcript analysis and fungal biomass quantification

For gene expression studies, fungal mycelia was grown in liquid CM for 48 hrs at 25 °C with agitation (150 rpm) before treatment with or without 5 mM H_2_O_2_ for 1hr (Figure 2A). Strains were also grown in CM for 48 hrs before switching to 1% (w/v) glucose minimal media (GMM) with either 10 mM sodium nitrate (ie. NADPH-replete media) or 10 mM ammonium tartrate as sole nitrogen sources for 16 hrs, following [31] (Figure 8A-C); or the mycelia was switched to nitrate minimal media with or without 1% (w/v) glucose for 16 hrs (Figure 8D). Mycelia was harvested, frozen in liquid nitrogen, and lyophilized for 24 hrs. A total of 100 mg of each mycelial sample was used to perform RNA extractions. RNA was extracted using the RNeasy Plant Mini Kit from Qiagen and treated with DNase I (Invitrogen) before conversion to cDNA using the qScript reagents from Quantas. The resulting cDNA was analyzed by quantitative real-time PCR (qPCR) in an Eppendorf Mastercycler® ep Realplex real-time PCR system. Reactions were performed using the 2X Quantifast® SYBR® Green PCR Master Mix (Qiagen) and the oligonucleotide primers listed in Table S2. Thermocycler conditions were: 5 min at 95°C initial denaturation, followed by 40 cycles of 95°C for 30 sec denaturation, 63°C for 30 sec annealing and 72°C for 1min extension.

The expression of each gene was normalized against the *M. oryzae* β-tubulin gene (*TUB2*). Results are given as the average of three technical replications and at least two biological replications. Results represent fold changes in gene expression, following normalization, between two strains (WT vs Δ*tps1*) or two growth conditions (CM vs CM + 10 mM H_2_O_2_; GMM + nitrate vs GMM + ammonium; or MM + glucose vs MM –glucose) obtained by the 2^−ΔΔCT^ method, as used previously [29], [31], [47]. Reciprocal values (ie Δ*tps1* vs WT) are also given to emphasize downregulated gene expression changes.

To determine the relative amount of fungal growth in infected leaves, DNA was extracted, in triplicate, from whole infected leaf tissues at 72 hpi using a HP fungal DNA mini Kit (Omega, BioTek). The relative amount of *M. oryzae* DNA was determined for each strain by qPCR using primers specific for the *M. oryzae* actin gene and normalized against the relative quantity of the rice actin gene.

## Supporting Information

Figure S1 **G6P sensing by Tps1 is required for antioxidation. (A)** We used our high-throughput gene disruption strategy [29] to generate an independent Δ*tps1* strain by replacing the *TPS1* coding region with *ILV1* conferring sulphonyl urea resistance. The resulting Δ*tps1::ILV1* strain was able to grow on 1% (w/v) glucose minimal media (GMM) containing ammonium as a sole nitrogen source. However, like the original hygromycin resistant Δ*tps1* strain [28], [31], the Δ*tps1::ILV1* strain was not able to utilize nitrate as a sole nitrogen source. The recapitulation of a nitrate non-utilizing phenotype confirms the loss of Tps1 function in the new Δ*tps1::ILV1* strain. Strains were grown on GMM [28], [31], [38] with the indicated sole nitrogen sources added at 10 mM final concentration. **(B)** Loss of Tps1 function in Δ*tps1* or Δ*tps1::ILV1* strains increases sensitivity to the oxidant menadione compared to WT. WT levels of resistance are restored in two Δ*tps1* strains expressing Tps1 proteins carrying the point-mutations R22G or Y99V in the G6P binding pocket of the active site. This suggests G6P sensing, but not Tps1 catalytic activity, is sufficient to restore resistance to oxidative stresses in Δ*tps1* strains. Strains were inoculated as 10 mm mycelial plugs onto 55 mm diameter plates of complete media (CM) containing menadione at the concentrations indicated. Images were taken after 5 days. NT =  no treatment.
(TIF)

Figure S2 **Δ** ***gtr1*** **strains are more sensitive to H_2_O_2_ and menadione than WT.** Disruption of the *GTR1* coding region resulted in increased sensitivity of Δ*gtr1* strains to H_2_O_2_ **(A)** and menadione **(B)** compared to WT strains on CM media. Compounds were added at the concentrations indicated. NT  =  no treatment.
(TIF)

Figure S3 **Impaired radial growth of thioredoxin mutant strains on H_2_O_2_ compared to WT.** WT, Δ*trr1* and Δ*tpx1* strains were grown on CM (*left panel*) and CM supplemented with 10 mM H_2_O_2_ (*right panel*). Strains were grown for 5 days, and radial diameters were measured. Δ*trr1* and Δ*tpx1* strains were significantly impaired (*Student’s t-test* p≤0.05) in radial growth compared to WT in the presence, but not absence, of 10 mM H_2_O_2_. Results are the average of three independent replicates. Error bars are standard deviation. Bars with the same letters are not significantly different (*Student’s t-test* p≤0.05). Measurements were taken after 5 days growth.
(TIF)

Figure S4 **Thioredoxin mutant strains Δ** ***trr1*** **and Δ** ***tpx1*** **form aberrant appressoria on rice cuticles but not artificial hydrophobic surfaces.** Axenic growth on CM was not impaired in *▵trr1* and *▵tpx1* strains compared to WT after 10 days (*left panel*). Spores of both *▵trr1* and *▵tpx1* strains, like those of WT, produced normal appressoria on artificial hydrophobic surface (plastic coverslips; *middle panel*). In contrast, on the leaf surface (*right panel*), *▵trr1* and *▵tpx1* strains developed aberrant appressoria (indicated by black arrows) and *▵tpx1* additionally produced unusual pigments in the conidia (indicated by white arrow). Scale bars: 10 µm.
(TIF)

Table S1 ***Magnaporthe oryzae*** **strains used in this study.**
(DOCX)

Table S2 **Oligonucleotide primers used in this study.**
(DOC)
