# Supplementary figures and images for: Characterizing Roles for the Glutathione Reductase, Thioredoxin Reductase and Thioredoxin Peroxidase-Encoding Genes of Magnaporthe oryzae during Rice Blast Disease
Source: PLoS One. 2014 Jan 24;9(1):e87300. doi: 10.1371/journal.pone.0087300 (PMC3901745; doi:10.1371/journal.pone.0087300)

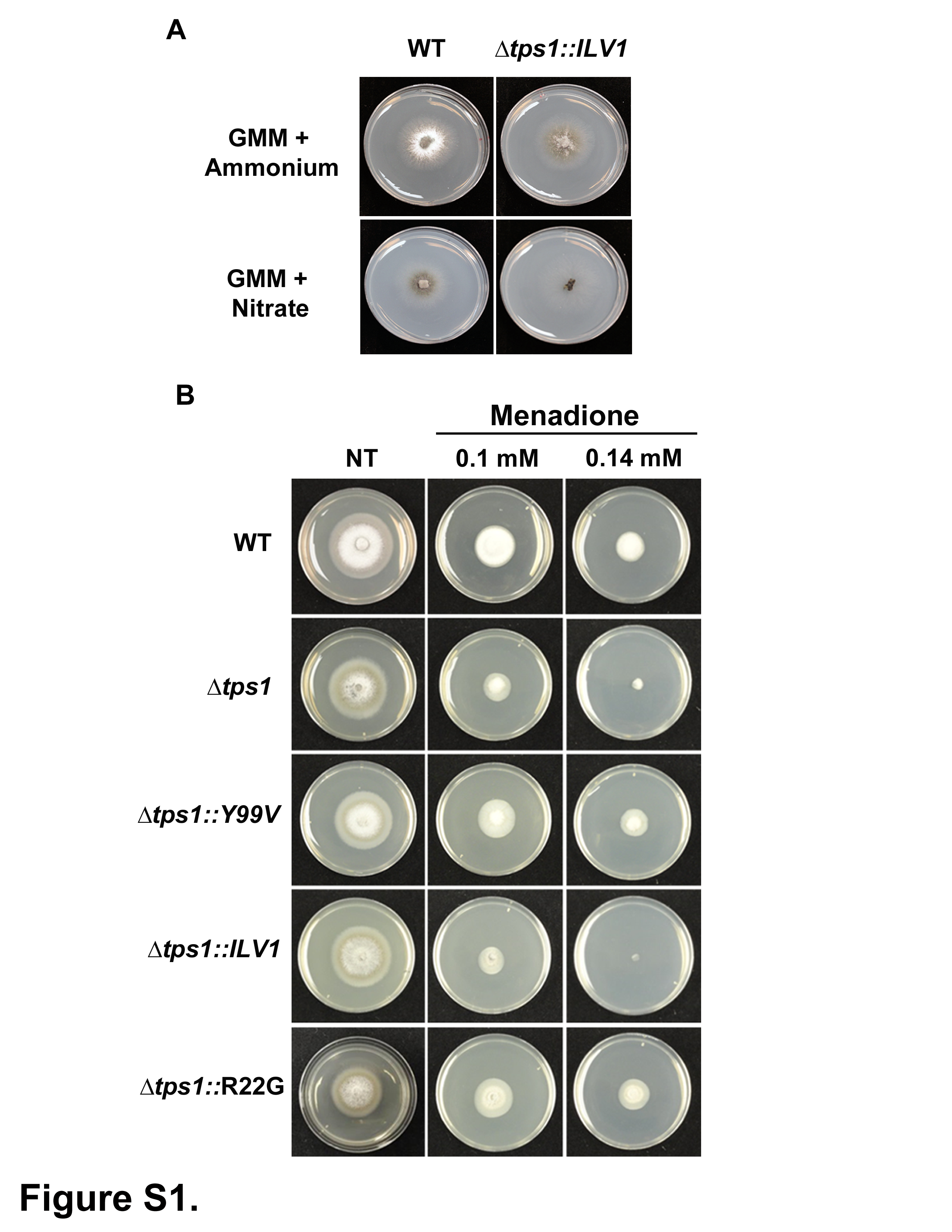

Supplement: Figure S1 — G6P sensing by Tps1 is required for antioxidation. (A) We used our high-throughput gene disruption strategy [29] to generate an independent Δtps1 strain by replacing the TPS1 coding region with ILV1 conferring sulphonyl urea resistance. The resulting Δtps1::ILV1 strain was able to grow on 1% (w/v) glucose minimal media (GMM) containing ammonium as a sole nitrogen source. However, like the original hygromycin resistant Δtps1 strain [28], [31], the Δtps1::ILV1 strain was not able to utilize nitrate as a sole nitrogen source. The recapitulation of a nitrate non-utilizing phenotype confirms the loss of Tps1 function in the new Δtps1::ILV1 strain. Strains were grown on GMM [28], [31], [38] with the indicated sole nitrogen sources added at 10 mM final concentration. (B) Loss of Tps1 function in Δtps1 or Δtps1::ILV1 strains increases sensitivity to the oxidant menadione compared to WT. WT levels of resistance are restored in two Δtps1 strains expressing Tps1 proteins carrying the point-mutations R22G or Y99V in the G6P binding pocket of the active site. This suggests G6P sensing, but not Tps1 catalytic activity, is sufficient to restore resistance to oxidative stresses in Δtps1 strains. Strains were inoculated as 10 mm mycelial plugs onto 55 mm diameter plates of complete media (CM) containing menadione at the concentrations indicated. Images were taken after 5 days. NT = no treatment. (TIF) [file pone.0087300.s001.tif]

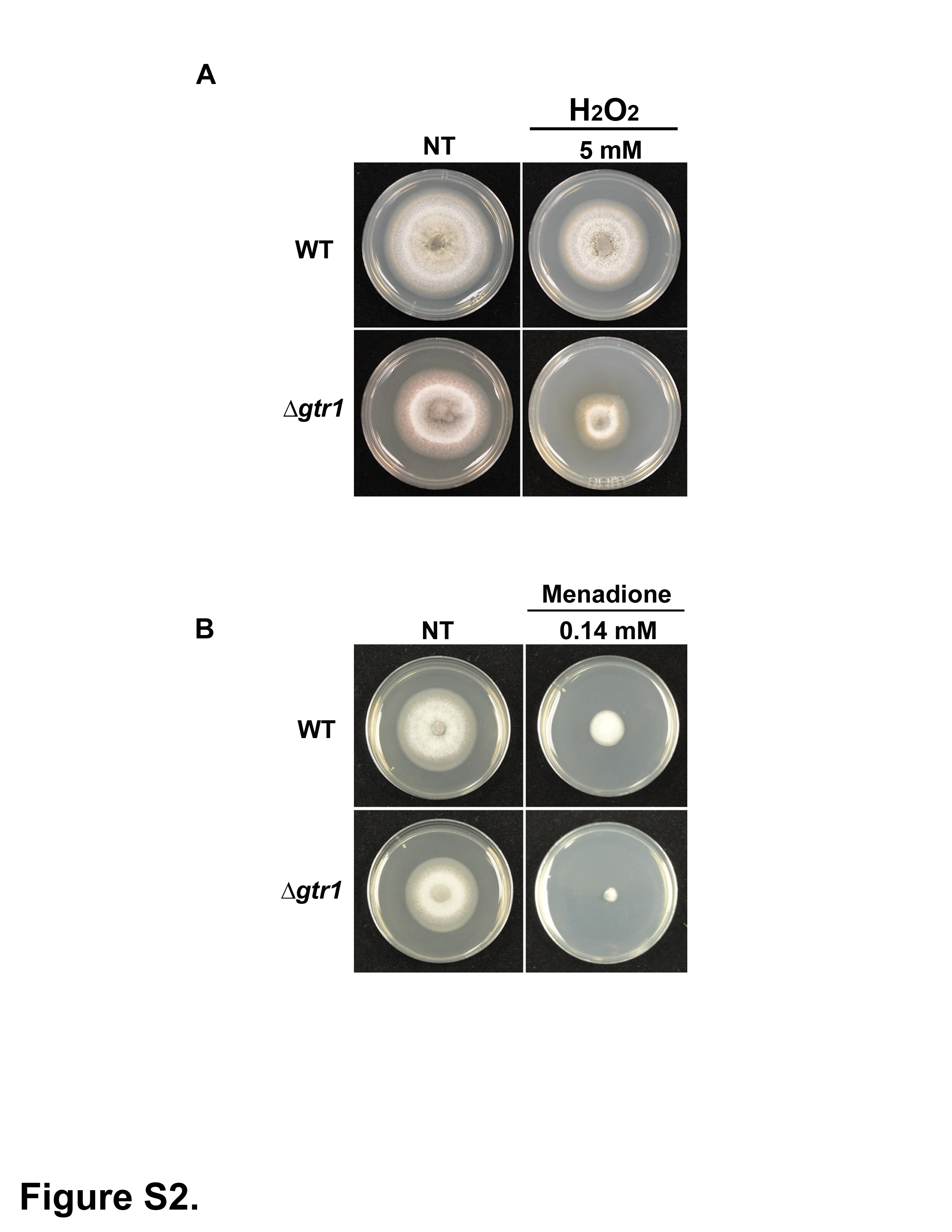

Supplement: Figure S2 — Δ gtr1 strains are more sensitive to H2O2 and menadione than WT. Disruption of the GTR1 coding region resulted in increased sensitivity of Δgtr1 strains to H2O2 (A) and menadione (B) compared to WT strains on CM media. Compounds were added at the concentrations indicated. NT = no treatment. (TIF) [file pone.0087300.s002.tif]

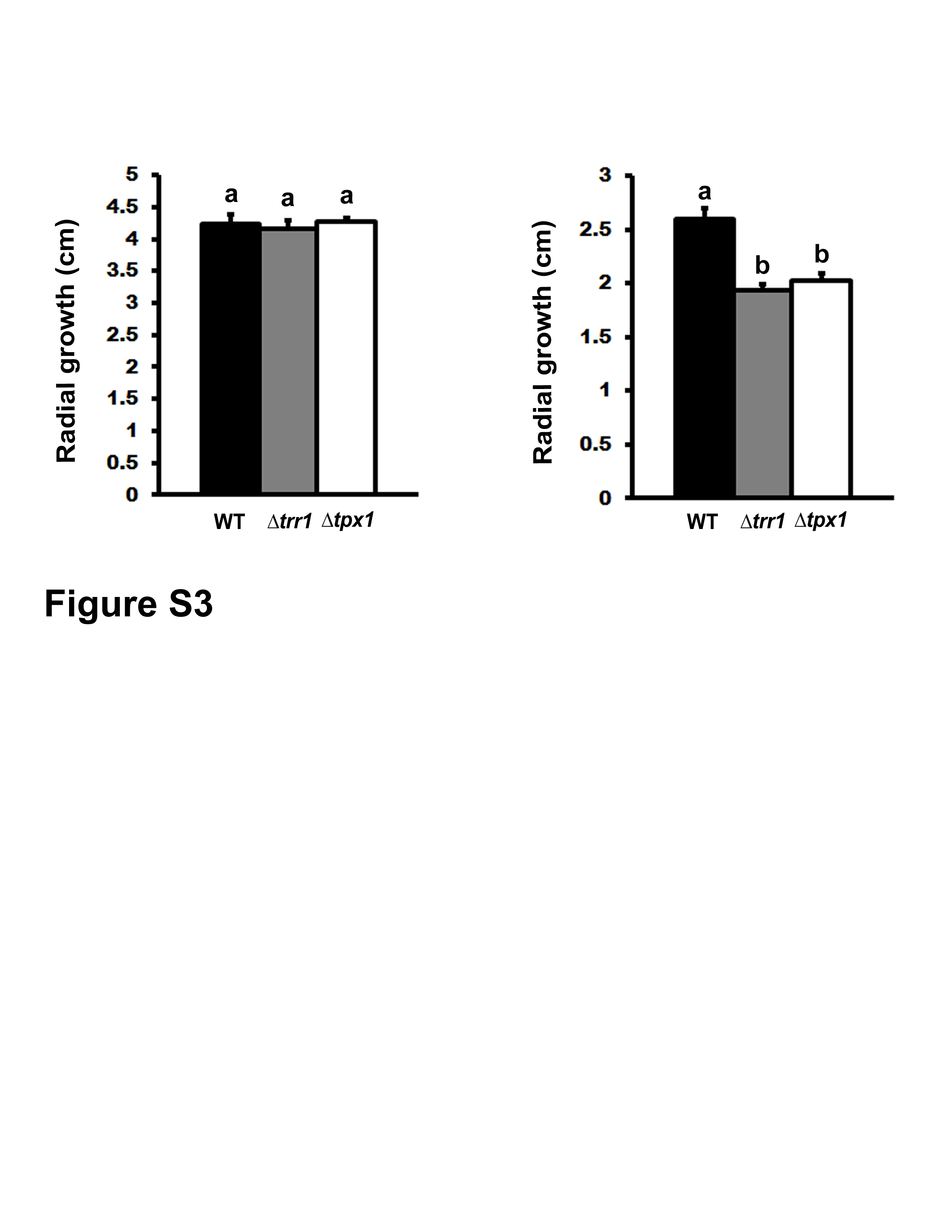

Supplement: Figure S3 — Impaired radial growth of thioredoxin mutant strains on H2O2 compared to WT. WT, Δtrr1 and Δtpx1 strains were grown on CM (left panel) and CM supplemented with 10 mM H2O2 (right panel). Strains were grown for 5 days, and radial diameters were measured. Δtrr1 and Δtpx1 strains were significantly impaired (Student’s t-test p≤0.05) in radial growth compared to WT in the presence, but not absence, of 10 mM H2O2. Results are the average of three independent replicates. Error bars are standard deviation. Bars with the same letters are not significantly different (Student’s t-test p≤0.05). Measurements were taken after 5 days growth. (TIF) [file pone.0087300.s003.tif]

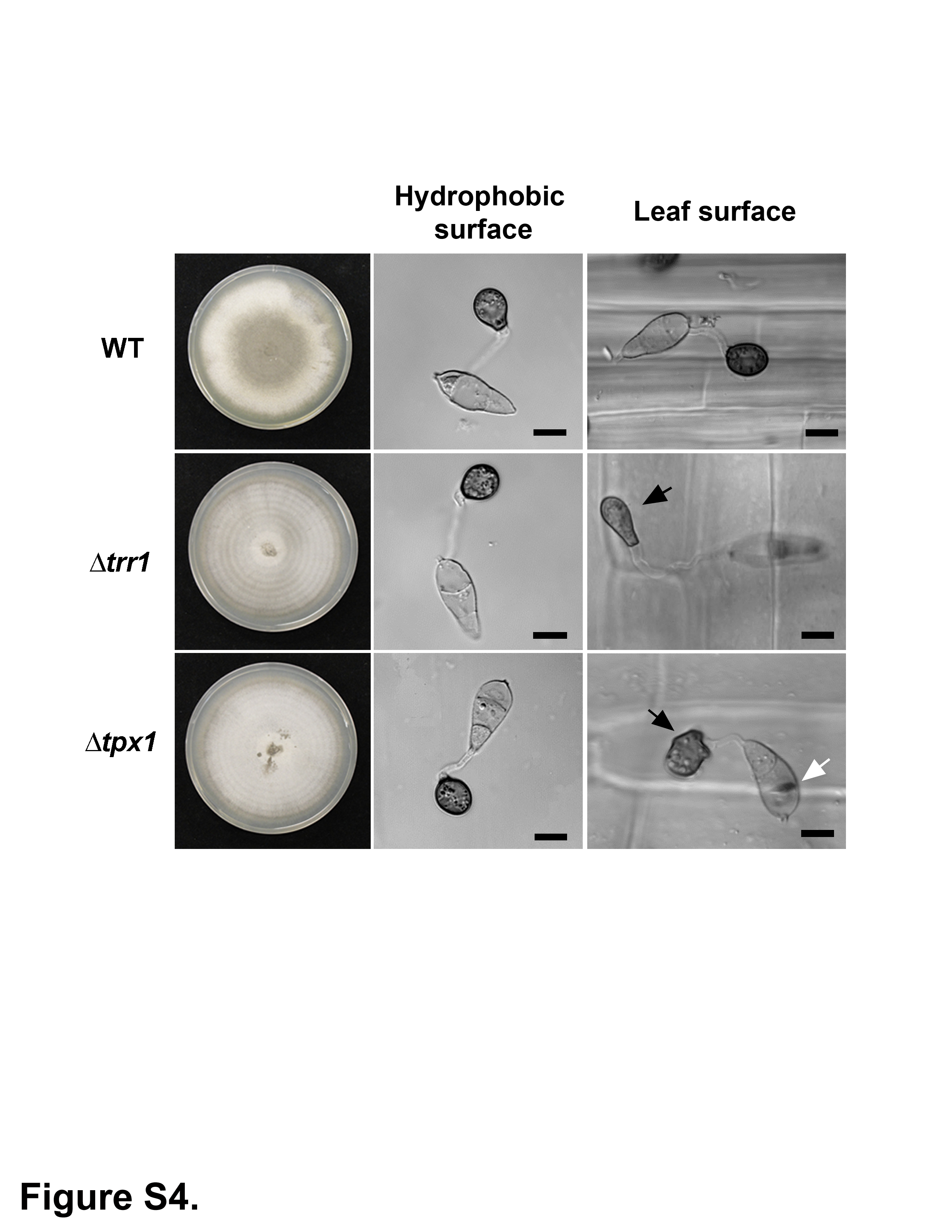

Supplement: Figure S4 — Thioredoxin mutant strains Δ trr1 and Δ tpx1 form aberrant appressoria on rice cuticles but not artificial hydrophobic surfaces. Axenic growth on CM was not impaired in ▵trr1 and ▵tpx1 strains compared to WT after 10 days (left panel). Spores of both ▵trr1 and ▵tpx1 strains, like those of WT, produced normal appressoria on artificial hydrophobic surface (plastic coverslips; middle panel). In contrast, on the leaf surface (right panel), ▵trr1 and ▵tpx1 strains developed aberrant appressoria (indicated by black arrows) and ▵tpx1 additionally produced unusual pigments in the conidia (indicated by white arrow). Scale bars: 10 µm. (TIF) [file pone.0087300.s004.tif]
