# Supplementary material for: Characterizing Roles for the Glutathione Reductase, Thioredoxin Reductase and Thioredoxin Peroxidase-Encoding Genes of Magnaporthe oryzae during Rice Blast Disease
Source: PLoS One. 2014 Jan 24;9(1):e87300. doi: 10.1371/journal.pone.0087300 (PMC3901745; doi:10.1371/journal.pone.0087300)
Supplement: Table S1 — Magnaporthe oryzae strains used in this study. (DOCX) [file pone.0087300.s005.docx]

**Table S1**. *Magnaporthe oryzae* strains used in this study.

| **Strains** | **Genotype** | **Reference** |
| --- | --- | --- |
| Guy11 | Wild type | [31] |
| Δ*gtr1* | Glutathione reductase (MGG_12749) deletion mutant of Guy1 | *This study* |
| Δ*trr1* | Thioredoxin reductase (MGG_01284) deletion mutant of Guy11 | *This study* |
| Δ*tpx1* | Thioredoxin peroxidase (MGG_07503) deletion mutant of Guy11 | *This study* |
| Δ*gtr1::GTR1* | Complementation strain resulting from integration of the full length *GTR1* gene and native promoter into the genome of strains carrying the Δ*gtr1* gene deletion. | *This study* |
| Δ*trr1::TRR1* | Complementation strain resulting from the integration of the full length *TRR1* gene and native promoter into the genome of strains carrying the Δ*trr1* gene deletion. | *This study* |
| Δ*txp1::TXP1* | Complementation strain resulting from the integration of the full length *TXP1* gene and native promoter into the genome of strains carrying the Δ*txp1* gene deletion. | *This study* |
| Δ*tps1* | Trehalose-6-phosphate synthase 1 (MGG_03860) deletion mutant of Guy11 constructed using *Hph* to confer hygromycin resistance. | [39] |
| Δ*tps1::ILV1* | Trehalose-6-phosphate synthase 1 (MGG_03860) deletion mutant of Guy11 constructed using *ILV1* to confer sulphonylurea resistance. | *This study* |
| Δ*tps1::R22G* | Complementation of Δ*tps1* with *TPS1* gene variant encoding the amino acid substitution R22G | [28, 31] |
| Δ*tps1::Y99V* | Complementation of Δ*tps1* with *TPS1* gene variant encoding the amino acid substitution Y99V | [28, 31] |
