# Supplementary material for: Characterizing Roles for the Glutathione Reductase, Thioredoxin Reductase and Thioredoxin Peroxidase-Encoding Genes of Magnaporthe oryzae during Rice Blast Disease
Source: PLoS One. 2014 Jan 24;9(1):e87300. doi: 10.1371/journal.pone.0087300 (PMC3901745; doi:10.1371/journal.pone.0087300)
Supplement: Table S2 — Oligonucleotide primers used in this study. (DOC) [file pone.0087300.s006.doc]

**Table S2**. Oligonucleotide primers used in this study.

| **Gene** | **Primer** | **Purpose** | **Sequence 5’ – 3’** |
| --- | --- | --- | --- |
| *ILV1* | M13F:IL | Sulphonylurea resistance gene amplification | CGCCAGGGGTTTTCCCAGTCACGACGTCGACGTGCCAACGCCACAG |
|  | ILSplit |  | AAGCATGTGCAGTGCCTTC |
|  | M13R:LV1 |  | AGCGGATAACAATTTCACACAGGAGTCGACGTGAGAGCATGCTAA |
|  | LV1Split |  | CGCCCGGCCGACATCC |
| *TPS1* (MGG_03860) | LF5’ | Split marker gene deletion | TCCAAGGATATTATTTTGCCGGTAA |
|  | LF3’ |  | GTCGTGACTGGGAAAACCCTGGCGCCAACAAAGTCCAGTAACGCAGG |
|  | RF5’ |  | TCCTGTGTGAAATTGTTATCCGCTGGCTTGACGGGGTAACATAATGACT |
|  | RF3’ |  | CCGGCGCGCTTCAACACT |
|  | NesF |  | TTTAAAACCCCCGCTTATACTTCG |
|  | NesR |  | CGGCCAGGAGGAGACGCAG |
| *GTR1* (MGG_12749) | LF5’ | Split marker gene deletion | GATACTTCGGGACCTTGCCAAGAT |
|  | LF3’ |  | GTCGTGACTGGGAAAACCCTGGCGCGACTCGTAGGCGTGATGGTGC |
|  | RF5’ |  | TCCTGTGTGAAATTGTTATCCGCTCAGTCGCTATCGCTGCCGGT |
|  | RF3’ |  | GTCAGTTTCTGGTCCAGATTCGGG |
|  | NesF |  | AAGGTGTGAATCAGGCTTAGGTATCAAT |
|  | NesR |  | CGTCCTTGGATACCTCGGGTTTT |
| *TRR1* (MGG_01284) | LF5’ | Split marker gene deletion | GTCAAGCTTGCTCGCAGGTCG |
|  | LF3’ |  | GTCGTGACTGGGAAAACCCTGGCGTTTGCGGCGGTCGATCTGA |
|  | RF5’ |  | TCCTGTGTGAAATTGTTATCCGCTGCGTTTTAGGTTGCACCGGTG |
|  | RF3’ |  | CATCTACTTCGTACACTCTTTTAGCGCC |
|  | NesF |  | GGACAATCTTCAAGATGTGACGGC |
|  | NesR |  | AATTCCCCCCATGCTCCCCC |
| *TPX1* (MGG_07503) | LF5’ | Split marker gene deletion | CGGAATCGTTTTCAAGACCAATCA |
|  | LF3’ |  | GTCGTGACTGGGAAAACCCTGGCGTGCGCGATTATGTGGGGAAGAT |
|  | RF5’ |  | TCCTGTGTGAAATTGTTATCCGCTTCTGGATTTATCAGCGATTGGCAC |
|  | RF3’ |  | CAGATCGCCCCCGGAATGTA |
|  | NesF |  | ATCGCAATTTTGAAGACATTCCTGA |
|  | NesR |  | CCAGATGCAGTGCGACGCCT |
| *GTR1* | qGTR1-F | Gene specific primers for qPCR | CGTGGGATTACACATTGTCGGC |
|  | qGTR1-R |  | TAGCTCCTCGGCACTCGTTGG |
| MGG_07317 | qGCL-F | Gene specific primers for qPCR | GGGCCAGAAATCAACCACGACC |
|  | qGCL-R |  | GCCCCTGAAGTTGCCGAGCA |
| MGG_06454 | qRTPCR-GS1-F | Gene specific primers for qPCR | CCTGCCTGTGGAACAACGCC |
|  | qRTPCR-GS1-R |  | AGGGCGACCGAGTCCATGC |
| *TRR1* | qTRR1-F | Gene specific primers for qPCR | CGTCGTTACTGGCAAGGAGGAGA |
|  | qTRR1-R |  | GGCTTAGTGACGATATAACCCTCCTCAT |
| *TPX1* | qTXP1-F | Gene specific primers for qPCR | AAGAAGGATAAGGAGGAGAAGGAGGC |
|  | qTXP1-R |  | CTATCCTTCTTAGCCTCCTTTTCGGTC |
| MGG_04236 | qTRX-F | Gene specific primers for qPCR | ATGCCGACATTCCTGCTCTTCAA |
|  | qTRX-R |  | TGACGACGGGTGGTTTGCG |
| *TUB1* (MGG_00604) | QRT-PCR b-tub F2 | Gene specific primers for qPCR | CGCGGCCTCAAGATGTCGT |
|  | QRT-PCR b-tub R2 |  | GCCTCCTCCTCGTACTCCTCTTCC |
| *MoACT1* (MGG_03982) | MgActin-U1 | Gene specific primers for qPCR | TCGACGTCCGAAAGGATCTGT |
|  | MgActin-L1 |  | ACTCCTGCTTCGAGATCCACATC |
| *OsACT2* | RiceACT-U1 | Gene specific primers for qPCR | CTGAAGAGCATCCTGTATTG |
|  | RiceACT-L1 |  | GAACCTTTCTGCTCCGATGG |
| *GTR1* | GTR1-GF | Gene-specific primers for complementation | TATAGGGCGAATTGGGTACTCAAATTGGTTTGGGATTCAGGGGAAGCAGATG |
|  | GTR1-GR |  | CCCGGTGAACAGCTCCTCGCCCTTGCTCACTAGCTCCTCGGCACTCGTTGG |
| *TRR1* | TRR1-GF | Gene-specific primers for complementation | TATAGGGCGAATTGGGTACTCAAATTGGTTTTTGGATGTTGTAGTCGGAAAGGGT |
|  | TRR1-GR |  | CCCGGTGAACAGCTCCTCGCCCTTGCTCACGGCTCGACGTCCTCTTGCTCAG |
| *TPX1* | TPX1-GF | Gene-specific primers for complementation | TATAGGGCGAATTGGGTACTCAAATTGGTTCGATTGGAGGGTTTGTTTCTGGAT |
|  | TPX1-GR |  | CCCGGTGAACAGCTCCTCGCCCTTGCTCACCTATCCTTCTTAGCCTCCTTTTCGGTC |
